# Itpka depletion implicates defects in anterior neural development of *Xenopus laevis*

**DOI:** 10.3389/fcell.2025.1610183

**Published:** 2025-07-09

**Authors:** Ernestine Saumweber, Marie-Christine Becker, Sophie Kunkel, Jana Welke, Sandra Schott, Christian Vizinho-Vieira, Selina Faoual, Michael J. Schmeisser, Susanne J. Kühl

**Affiliations:** ^1^ Institute of Biochemistry and Molecular Biology, Ulm University, Ulm, Germany; ^2^ Institute of Anatomy, University Medical Center of the Johannes Gutenberg-University Mainz, Mainz, Germany; ^3^ Focus Program Translational Neurosciences, University Medical Center of the Johannes Gutenberg-University Mainz, Mainz, Germany

**Keywords:** ITPKA, anterior neural development, *Xenopus laevis*, disease modeling, embryogenesis

## Abstract

Inositol 1,4,5-trisphosphate 3-kinase A (Itpka) is a neuronal isoform of the ITPK family that regulates both actin dynamics and calcium signaling. While *itpka* deficiency in adult mice mainly results in central nervous system phenotypes, its contribution to early development remains unclear. To study the role of Itpka in embryogenesis, we used the South African clawed frog, *Xenopus laevis,* as vertebrate model organism. Our analysis revealed that *itpka* is specifically expressed in distinct regions of the developing anterior neural tissue. To investigate Itpka function during early anterior neural development, we generated a morpholino oligonucleotide (MO)-mediated *itpka* knockdown approach. The depletion of Itpka leads to defects in head, brain, and eye development which can be rescued by *Xenopus itpka* RNA co-injection. An analysis of the underlying molecular basis revealed a reduced expression of key genes associated with head, brain and eye development in Itpka MO-injected embryos. These findings highlight a crucial role of Itpka during anterior neural development in *Xenopus laevis* and indicate that the function of Itpka needs to be further investigated.

## 1 Introduction

The family of Inositol 1,4,5-trisphosphate 3-kinase (ITPKs) includes ITPKA, ITPKB and ITPKC. ITPKA was found to be the neuronal isoform (Schell, 2010) and its activity was first measured in the developing rat cerebral cortex (Heacock et al., 1990). *Itpka* could slightly been detected during mouse development via northern blot analysis and is mainly expressed in the adult brain (Mailleux et al., 1993; Vanweyenberg et al., 1995). Herein, *Itpka* has predominately been detected in pyramidal cells of the hippocampal CA1 region and granule cells of the dentate gyrus as well as in Purkinje cells of the cerebellum (Mailleux et al., 1993). On a subcellular level, ITPKA is localized at postsynaptic densities (PSDs) in mature neurons, where the protein is bound to F-actin (Schell et al., 2001; Windhorst et al., 2012; Yamada et al., 1993). This interaction regulates the number and morphology of hippocampal dendritic spines, as well as the number of synapses (Ashour et al., 2015; Johnson and Schell, 2009; Kim et al., 2009; Köster et al., 2016). *Itpka* deficiency in mice impairs the formation of synaptic contacts, decreases synaptic strength and disrupts spatial memory (Jun et al., 1998; Kim et al., 2009; Köster et al., 2016).

Besides its function in actin bundling, Itpka is also involved in calcium signaling which controls many different cellular processes such as metabolism, cell proliferation and brain development (Berridge, 2016). Thereby, Itpka phosphorylates the second messenger inositol 1,4,5-trisphosphate (InsP_3_) to inositol 1,3,4,5-tetrakisphosphate (InsP_4_) and modulates InsP_3_-dependent calcium release (Berridge, 2016; Windhorst et al., 2012). Recent findings also demonstrated that *Itpka* depleted mice show impairments in energy metabolism (Blechner et al., 2020).

However, the molecular mechanism controlling *itpka* regulation has not yet been fully clarified and its role during early embryonic development has hardly been investigated. Since *Xenopus laevis* (*X. laevis*) is an established and well described model organism for embryonic development, we used it for our study. We found that *itpka* is primarily expressed in distinct regions of anterior neural tissue in *X. laevis* during early development. Depletion of Itpka resulted in abnormal head, brain and eye development of *X. laevis* embryos. These phenotypes were significantly rescued by the co-injection of *X. laevis itpka* RNA. Additionally, Itpka knockdown (KD) led to a reduced expression of several important genes for proper anterior neural development.

## 2 Results

### 2.1 Itpka is evolutionary highly conserved across different species and expressed in anterior neural tissue during *X. laevis* embryogenesis

**FIGURE 1:**
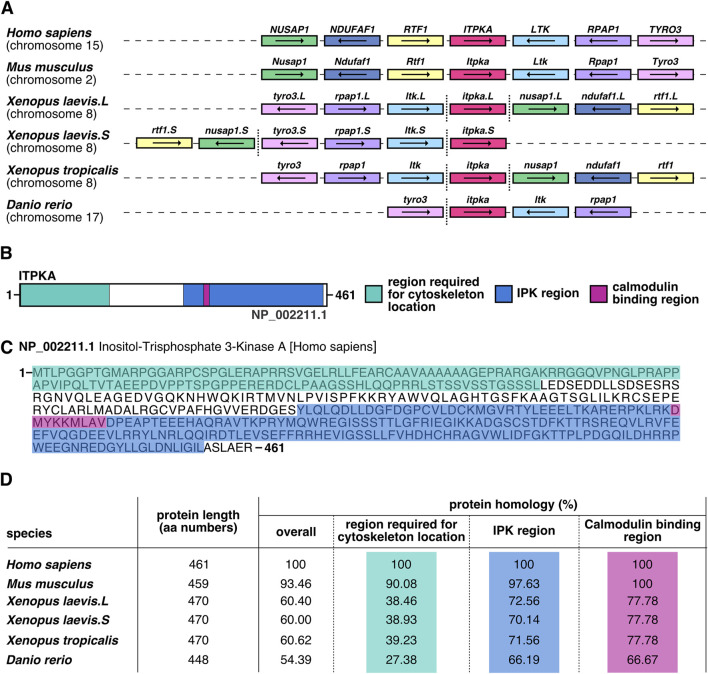
*In silico* analysis of *itpka* reveals a strong conservation across species. **(A)** Synteny analysis of *itpka* genes’ location and its neighboring genes among different species such as *Homo sapiens*, *Mus musculus*, *Xenopus laevis*, *Xenopus tropicalis* and *Danio rerio*. Conserved genes are depicted by boxes with identical colors, *itpka* is shown in red. Non-conserved neighboring genes are not shown. The orientations of the open reading frames are indicated by arrows. Gene lengths and the distances are not proportional to their actual size. More distanced genes on the same chromosome are emphasized by a vertical dashed line. Chromosomal location is listed below the species name. *X. laevis* L or S chromosome is specified next to the gene name. **(B)** Schematic representation of human ITPKA. Region required for cytoskeleton location (green), IPK region (blue) and calmodulin binding region (pink) are shown. **(C)** Human ITPKA protein sequence. Amino acid (aa) sequence is shown. The region required for cytoskeleton location (green), IPK region (blue) and calmodulin binding region (pink) are marked. **(D)** Homology of the aa sequences of full-length ITPKA among different species. Aa length is given in numbers. Percentages represent identical residues (percent identity) of the indicated species compared to *Homo sapiens*. Abbreviation: aa, amino acid; IPK, inositol polyphosphate kinases; *itpka*, *inositol 1,4,5-trisphosphate 3-kinase A*; *LTK, leukocyte receptor tyrosine kinase*; *NDUFAF1, NADH:ubiquinone oxidoreductase complex assembly factor 1*; *NUSAP1, nucleolar and spindle associated protein 1*; *RPAP1, RNA polymerase II associated protein 1*; RTF1, *RTF1 homolog, Paf1/RNA polymerase II complex component*; TYRO3, *TYRO3 protein tyrosine kinase*.

To get insights into the conservation of *itpka* gene location and Itpka protein homology of *X. laevis* in comparison to *Homo sapiens*, *in silico* analysis was performed. Comparative synteny analysis revealed that the location of *itpka* and the surrounding genes on the chromosome are highly conserved across species (Figure 1A). A schematic overview of the ITPKA protein illustrates the region required for cytoskeleton location, the inositol polyphosphate kinase (IPK) region as well as calmodulin binding region (Figures 1B,C). The full-length protein of human ITPKA demonstrated comparable amino acid lengths and a high evolutionary conservation of the protein sequences across different species (Figure 1D).

**FIGURE 2:**
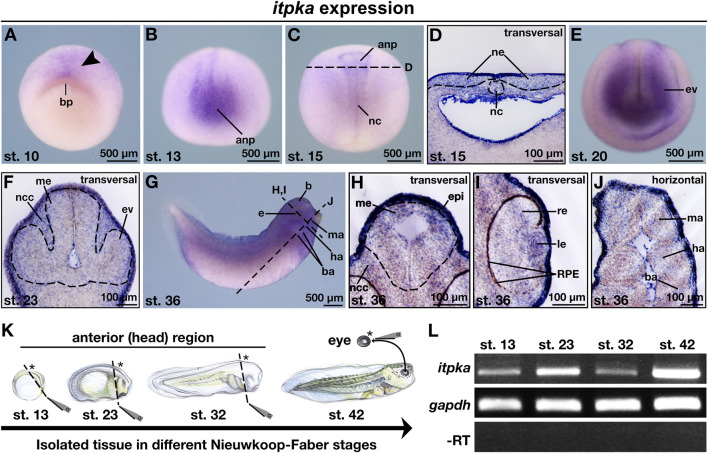
*Itpka* is expressed during *X. laevis* anterior neural development. **(A–J)** Spatio-temporal expression pattern of *itpka* visualized by whole mount *in situ* hybridization (WMISH) during *X. laevis* development. Embryonic stages and scale bars are indicated in each panel. Black dashed lines represent section planes. **(A)** Animal view of *X. laevis* at stage 10. **(B)** Anterior and **(C)** dorsal view of embryos at stage 13 and 15. **(D)** Transversal section of stage 15. **(E)** Anterior view of a *X. laevis* embryo at stage 20. **(F)** Transversal section at stage 23. **(G)** Lateral view. **(H,I)** Transversal sections and **(J)** horizontal section at stage 36. **(K)** Illustration of the isolated tissue (marked with asterisk) of the anterior (head) structures and the eye in different Nieuwkoop-Faber stages (st. 13, 23, 32, 42). **(L)** Temporal expression pattern of *itpka* during *X. laevis* anterior neural development analyzed by semi-quantitative reverse transcriptase (RT)-PCR with *X. laevis* cDNA templates of the indicated stages and isolated tissues. All three genes were detected in all investigated stages. *Gapdh* was used as loading control, and as negative control -RT which reaction lacks reverse transcriptase. Abbreviations: anp, anterior neural plate; b, brain; ba, branchial arch; bp, blastopore; cDNA, copyDNA; e, eye; epi, epidermis; ev, eye vesicle; *gapdh, glyceraldehyde 3-phosphate dehydrogenase*; ha, hyoid arch; le, lens; ma, mandibular arch; me, mesencephalon; µm, micrometer; nc, notochord; ncc, neural crest cells; *itpka*, *inositol 1,4,5-trisphosphate 3-kinase A*; PCR, polymerase chain reaction; re, retina; RPE, retinal pigmented epithelium; RT, reverse transcriptase; st., stage; WMISH, whole mount *in situ* hybridization.

The knowledge of the spatio-temporal expression pattern of *itpka* during the early development of vertebrates such as *X. laevis* remains unknown so far. To provide a detailed expression pattern analysis of *itpka* during the development of *X. laevis*, we used the whole mount *in situ* hybridization (WMISH) technique. First, we have designed a sense and an antisense probe to validate if the *itpka* digoxigenin-labeled antisense probe binds specifically to the *X. laevis itpka* mRNA in comparison to the digoxigenin-labeled sense probe. Expression analysis revealed a specific expression pattern of the *itpka* antisense probe and thus we used it for the detailed analysis (Supplementary Figure S1). At early cleavage stages, *itpka* transcripts were detected in the neuroectoderm next to the blastopore of *X. laevis* embryos (Figure 2A; arrowhead). At stage 13, *itpka*-positive cells were found in the anterior neural tissue (Figure 2B). At stage 15, *itpka* is expressed in a distinct region in the anterior neural plate and the notochord (Figures 2C,D). From stage 20 onward, we found *itpka* transcripts predominantly in the developing eye, brain, epidermis and migrating neural crest cells (NCCs) such as the mandibular (ma), hyoid (ha), and branchial arches (ba) (Figures 2E–J). The RT-PCR analyses using isolated tissues of different stages (Figure 2K) revealed an expression of *itpka* in the anterior (head) region and the eye (Figure 2L).

### 2.2 Itpka knockdown results in a severe head and eye phenotype

Our data indicated that during early *X. laevis* development, *itpka* transcripts were enriched in developing anterior neural tissue (Figure 2). We thus introduced an antisense-based morpholino oligonucleotide (MO) KD approach to investigate the possible effects of Itpka depletion in anterior neural development of *X. laevis*. First of all, we investigated whether the Itpka MO efficiently binds to the *X. laevis itpka* MO binding site. Therefore, the respective MO binding site of *X. laevis* (*Xitpka MObs*) and the corresponding *Δ 5′UTR X. laevis itpka* MO binding site (*Δ 5′UTR XitpkaMObs*) were cloned in front of and in frame with green fluorescent protein (*GFP*). RNA of these constructs was co-injected together with Itpka MO and control morpholino oligonucleotide (CoMO) into two-cell stage embryos. GFP expression was then monitored in stage 20. Itpka MO blocked GFP expression upon *Xitpka MObs-GFP* RNA co-injection, whereas CoMO did not, indicating the interference of Itpka MO with *Xitpka MObs-GFP* translation. In addition, Itpka MO did not block translation of *Δ 5′UTR XitpkaMObs-GFP* demonstrating that RNA coding for *Δ 5′UTR itpka* RNA is suitable for rescue experiments (Supplementary Figure S2).

**FIGURE 3:**
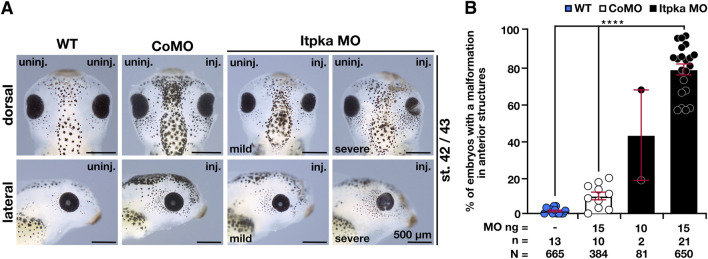
Itpka MO knockdown results in defects in anterior neural development in a dose-dependent manner. **(A)** The dorsal and lateral views of the embryo show the head and eye phenotype at stage 42/43. Representative embryos are shown. **(B)** Statistical evaluation of smaller and deformed heads and eyes as indicated in **(A)**. Abbreviations: CoMO, control morpholino oligonucleotide; inj., injected; *itpka*, *inositol 1,4,5-trisphosphate 3-kinase A;* µm, micrometer; MO, morpholino oligonucleotide; n, number of independent experiments; N, number of analyzed embryos in total; ng, nanogram; st., stage; uninj., uninjected; WT, wildtype. Error bars indicate standard errors of the means: ****, p ≤ 0.0001.

To investigate the Itpka MO KD, we injected Itpka MO unilaterally into one animal dorsal blastomere of *X. laevis* embryos at the eight-cell stage that gives rise to the anterior neural tissue (Moody and Kline, 1990). To monitor the targeted region, we co-injected 0.4–0.5 ng GFP RNA and sorted the embryos according to its specific expression in the anterior neural tissue. CoMO that cannot bind to any *X. laevis* mRNA was used as injection control (Eisen and Smith, 2008). Itpka MO-injected embryos exhibited a spectrum of anomalies ranging from significantly smaller and/or deformed heads or eyes on the injected side to a lack of anterior head structures in an Itpka MO-dose dependent manner (Figures 3A,B).

### 2.3 Itpka is required for head development in *X. laevis*

**FIGURE 4:**
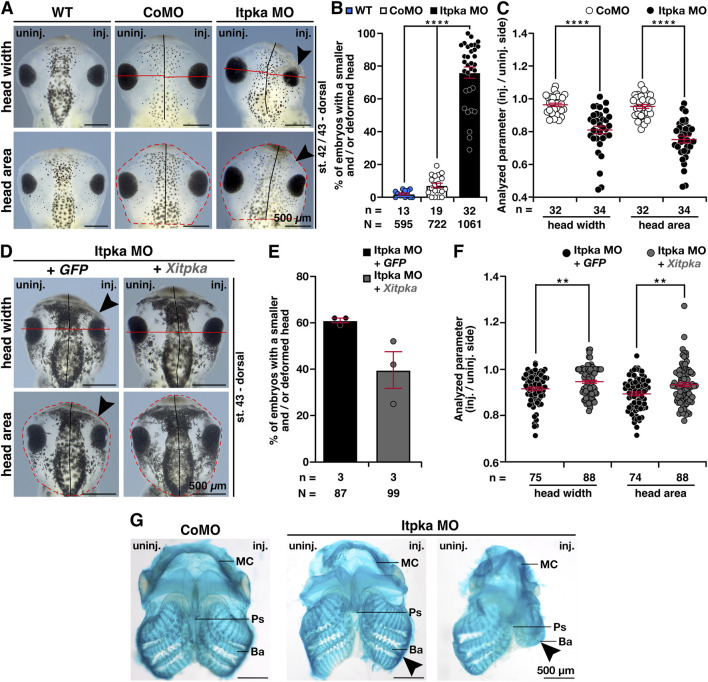
Itpka MO injection leads to a severe head phenotype that is rescued by *Xitpka* RNA. **(A)** The dorsal views of representative embryos at stage 42/43 are shown. Red lines indicate the measured head width and area. **(B)** Statistical evaluation of smaller and/or deformed heads between wildtype/CoMO-injected embryos compared to Itpka MO-injected embryos. **(C)** Statistical evaluation of the head width and area as indicated in **(A)**. **(D)** Co-injection of *Xenopus Itpka*-RNA. The dorsal views of representative stage 43 embryos are shown. Red lines indicate the measured width and area. **(E)** Statistical evaluation of smaller and/or deformed heads between Itpka MO-injected embryos compared to Itpka MO-injected embryos with *Xitpka* co-injection. **(F)** Statistical evaluation of the head width and area as illustrated in **(D)**. **(G)** Ventral view of Alcian blue-stained and dissected cranial cartilages from control and Itpka morphants. Deformed cartilage structures are shown by black arrowheads, especially at the Meckel’s cartilage (MC) and branchial arch (ba). Abbreviations: Ba, Branchial arch; CoMO, control morpholino oligonucleotide; GFP, green fluorescent protein; inj., injected; *itpka*, *inositol 1,4,5-trisphosphate 3-kinase A*; MC, Meckel’s cartilage; µm, micrometer; MO, morpholino oligonucleotide; n, number of independent experiments; N, number of analyzed embryos in total; Ps, Parasphenoid; st., stage; uninj., uninjected; WT, wildtype, *Xitpka*, *Xenopus itpka*. Error bars indicate standard errors of the means. **, p ≤ 0.01; ****, p ≤ 0.0001.

**FIGURE 5:**
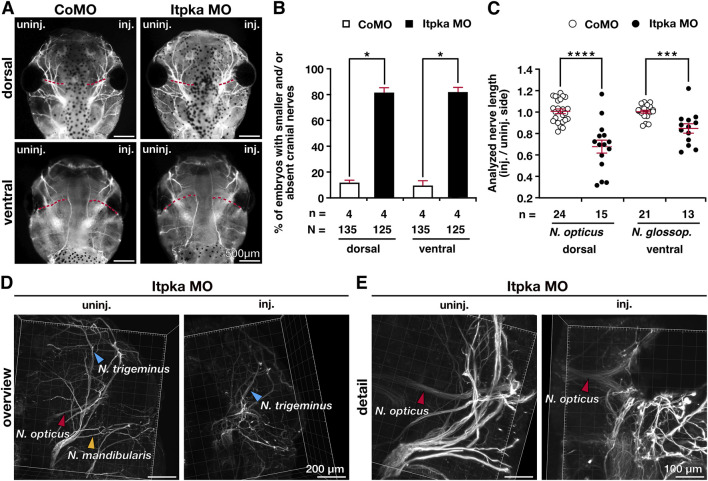
Itpka knockdown results in malformed cranial nerves. **(A)** Dorsal and ventral views of CoMO- and Itpka MO-injected embryos show the branching of cranial nerves upon Itpka MO KD visualized by 3A10 antibody staining. **(B)** Statistical evaluation of embryos with shortened or absent branching of cranial nerves as illustrated in **(A)**. **(C)** Statistical analysis of cranial nerve length of the *Nervus opticus* (*N. opticus*) and the *Nervus glossopharyngeus* (*N. glossop.*) illustrated in **(A)**. **(D,E)** Confocal images of an Itpka MO-injected embryo are shown. An overview **(D)** of the stained *Nervus opticus (N. opticus)*, *Nervus trigeminus (N. trigeminus)* and *Nervus mandibularis (N. mandibularis)* and a detailed view **(E)** of the stained *Nervus opticus (N. opticus)*. Abbreviations: CoMO, control morpholino oligonucleotide; inj., injected; *itpka*, *inositol 1,4,5-trisphosphate 3-kinase A*; KD, knockdown; µm, micrometer; MO, morpholino oligonucleotide; st., stage; uninj., uninjected. Error bars indicate standard errors of the means. *, p ≤ 0.05; ***, p ≤ 0.001; ****, p ≤ 0.0001.

To analyze the head phenotype upon Itpka MO KD in more detail, we first quantified the embryos revealing significantly more embryos with smaller and/or deformed head compared to wildtype (WT) and CoMO-injected embryos (Figures 4A,B). The measurement of head width and area of *X. laevis* embryos showed significantly smaller heads (Figures 4A,C). Co-injection of *X. laevis itpka* (*Xitpka*) RNA that is not targeted by Itpka MO significantly rescued the Itpka MO-induced head width and area indicating the specificity of Itpka MO-induced phenotypes (Figures 4D–F). The smaller heads of Itpka morphants prompted us to focus more on the cartilage. Alcian blue staining at late tadpole stages showed a reduction of branchial arch and Meckel’s cartilage in Itpka MO-injected embryos (Figure 4G). In addition, 3A10 antibody staining revealed a clear shortening and/or disorganization of the cranial nerves upon Itpka KD (Figures 5A,B). For instance, the *Nervus opticus (N. opticus)* and *Nervus glossopharyngeus (N. glossop.)* were shortened in length (Figures 5A,C). Furthermore, confocal images revealed a more diffuse organization of the *N. opticus*, *Nervus trigeminus (N. trigeminus)* and *Nervus mandibularis (N. mandibularis)* (Figures 5D,E).

After observing smaller head structures in Itpka MO-induced embryos, we thought that a possible reason of smaller heads could be fewer proliferative cells or more apoptotic cells upon Itpka KD, but neither pH H3 nor TUNEL staining was altered in the anterior neural region of the head compared to the control embryos at stage 23 (Supplementary Figure S3). These results indicate that the observed changes are not due to altered or abnormal proliferation or apoptosis in the respective area and at this early stage in development.

**FIGURE 6:**
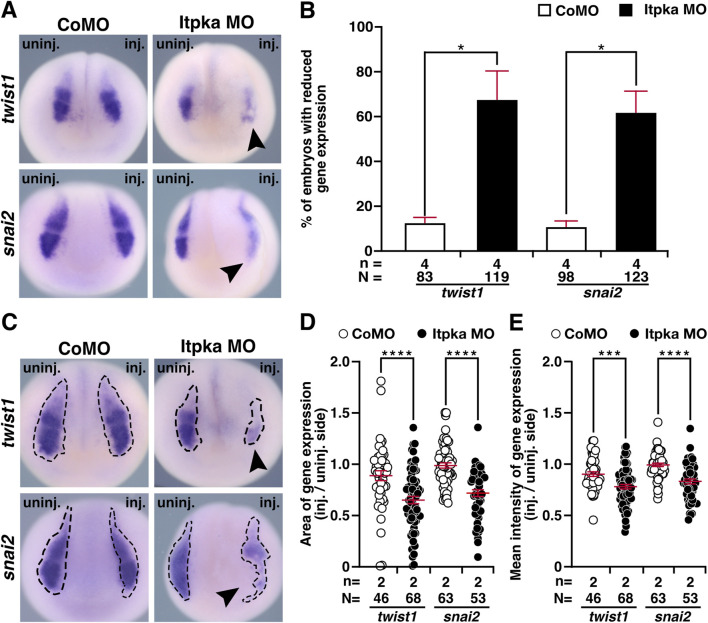
Itpka MO injection impairs neural crest cell induction and migration at stage 15. **(A)** Anterior view of CoMO- and Itpka MO-injected embryos at stage 15 after whole mount *in situ* hybridization (WMISH) with NCC-specific genes *twist1* and *snai2* during NCC induction and migration. Reduced gene expression on the Itpka MO-injected side is indicated by black arrowheads. **(B)** Statistical evaluation of NCC-specific gene expression as illustrated in **(A)**. **(C)** Anterior view of CoMO- and Itpka MO-injected embryos at stage 15. The analyzed gene expression area and the area of the measured mean intensity of gene expression of *twist1* and *snai2* is marked with a dashed line. Reduced gene expression on the Itpka MO-injected side is indicated by black arrowheads. **(D)** Statistical evaluation of the gene expression area of *twist1* and *snai2* as illustrated in **(C)**. **(E)** Statistical evaluation of the mean intensity of gene expression of *twist1* and *snai2* as illustrated in **(C)**. Abbreviations: CoMO, control morpholino oligonucleotide; inj., injected; MO, morpholino oligonucleotide; n, number of independent experiments; N, number of analyzed embryos in total; *itpka*, *inositol 1,4,5-trisphosphate 3-kinase A*; *snai2*, snail family transcriptional repressor 2; st., stage; *twist1*, twist family bHLH transcription factor 1; uninj., uninjected; WMISH, whole mount *in situ* hybridization. Error bars indicate standard errors of the means: *, p ≤ 0.05; ***, p ≤ 0.001; ****, p ≤ 0.0001.

**FIGURE 7:**
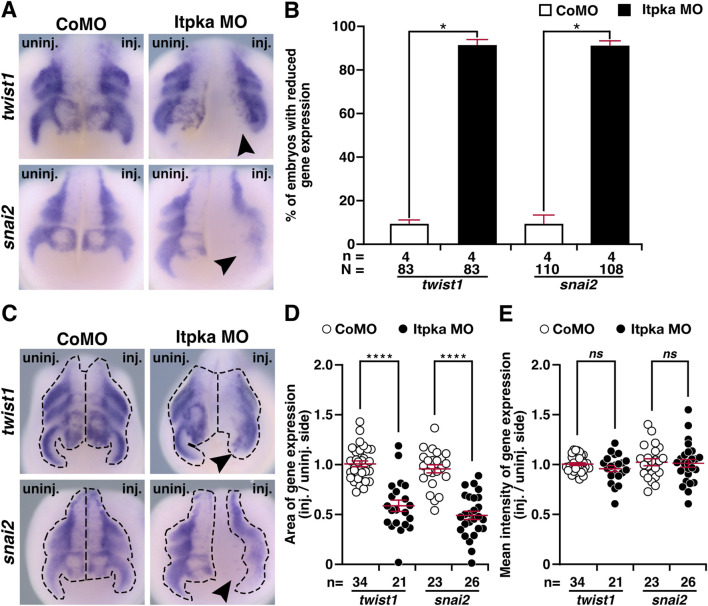
Itpka MO knockdown influences migration of neural crest cells at stage 20. **(A)** Anterior view of CoMO- and Itpka MO-injected embryos at stage 20 after whole mount *in situ* hybridization (WMISH) with NCC-specific genes during NCC migration such as *twist1* and *snai2*. Reduced gene expression on the Itpka MO-injected side is indicated by black arrowheads. **(B)** Statistical evaluation of NCC-specific gene expression as illustrated in **(A)**. **(C)** Anterior view of CoMO- and Itpka MO-injected embryos at stage 20. The analyzed gene expression area and the area of the measured mean intensity of gene expression of *twist1* and *snai2* is marked with a dashed line. Reduced gene expression on the Itpka MO-injected side is indicated by black arrowheads. **(D)** Statistical evaluation of the gene expression area of *twist1* and *snai2* as illustrated in **(C)**. **(E)** Statistical evaluation of the mean intensity of gene expression of *twist1* and *snai2* as illustrated in **(C)**. Abbreviations: CoMO, control morpholino oligonucleotide; inj., injected; MO, morpholino oligonucleotide; n, number of independent experiments; N, number of analyzed embryos in total; *ns*, non-significant; *itpka*, *inositol 1,4,5-trisphosphate 3-kinase A*; *snai2*, snail family transcriptional repressor 2; st., stage; *twist1*, twist family bHLH transcription factor 1; uninj., uninjected; WMISH, whole mount *in situ* hybridization. Error bars indicate standard errors of the means: *ns*, p > 0.05; *, p ≤ 0.05; ****, p ≤ 0.0001.

**FIGURE 8:**
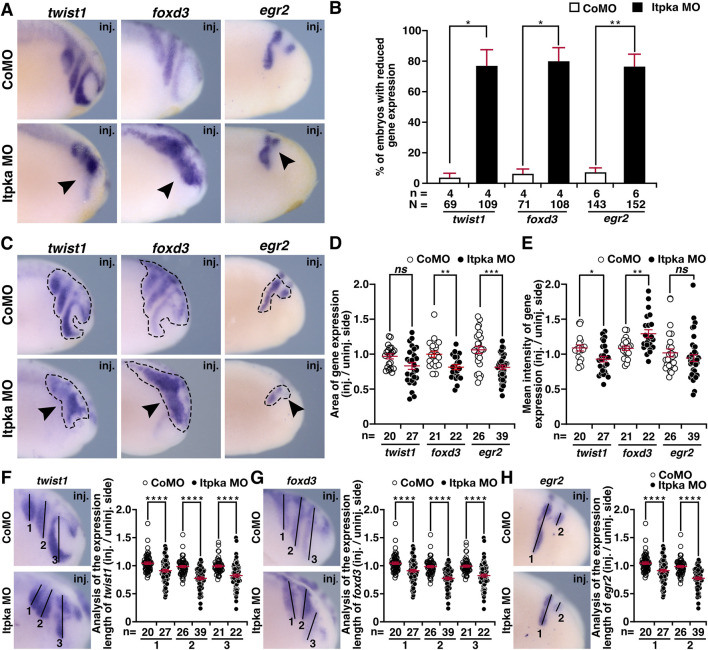
Itpka MO injection hinders proper migration of neural crest cells at stage 23. **(A)** Lateral view of CoMO- and Itpka MO-injected embryos at stage 23 after whole mount *in situ* hybridization (WMISH) with NCC-specific genes during NCC migration *twist1, foxd3* and *egr2*. Reduced gene expression on Itpka MO-injected side is indicated by black arrowheads. **(B)** Statistical evaluation of NCC-specific gene expression as illustrated in **(A)**. **(C)** Lateral view of CoMO- and Itpka MO-injected embryos at stage 23. The analyzed gene expression area and the area of the measured mean intensity of gene expression of *twist1, foxd3* and *egr2* is marked with a dashed line. Reduced gene expression on Itpka MO-injected side is indicated by black arrowheads. **(D)** Statistical evaluation of the gene expression area of *twist1, foxd3* and *egr2* as illustrated in **(C)**. **(E)** Statistical evaluation of the mean intensity of gene expression of *twist1, foxd3* and *egr2* as illustrated in **(C)**. **(F)** Lateral view of CoMO- and Itpka MO-injected embryos at stage 23 showing the analyzed length (1,2,3) of *twist1* gene expression and statistical evaluation of the length of *twist* expression (1,2,3). **(G)** Lateral view of CoMO- and Itpka MO-injected embryos at stage 23 showing the analyzed gene expression length (1,2,3) of *foxd3* and the statistical evaluation of NCC-specific genes’ expression length. **(H)** Lateral view of CoMO- and Itpka MO-injected embryos at stage 23, showing the analyzed *egr2* gene expression length (1,2) and the statistical evaluation of *egr2* expression length (1,2) in detail. Abbreviations: CoMO, control morpholino oligonucleotide; *egr2*, early growth response 2; *foxd3*, forkhead box D3; inj., injected; MO, morpholino oligonucleotide; n, number of independent experiments; N, number of analyzed embryos in total; *ns*, non-significant; *itpka*, *inositol 1,4,5-trisphosphate 3-kinase A*; st., stage; *twist1*, twist family bHLH transcription factor 1; WMISH, whole mount *in situ* hybridization. Error bars indicate standard errors of the means: *ns*, p > 0.05; *, p ≤ 0.05; **, p ≤ 0.01; ***, p ≤ 0.001; ****, p ≤ 0.0001.

Since *itpka* is expressed in NCCs (Figures 2F–H,J), and Itpka KD led to deformed NCC derivates such as cranial cartilage and nerves, we investigated the NCC-specific genes *twist1* (*twist family bHLH transcription factor 1*), *snai2* (snail family transcriptional repressor 2), *foxd3* (*forkhead box D3*) and *egr2* (*early growth response 2*) by WMISH. At stage 15, the neurula stage, the induction of the neural crest cells is initiated. At this stage expression of *twist1* and *snai2* was strongly reduced upon Itpka KD (black arrowheads) (Figures 6A,B). Detailed analysis revealed that the expression area and intensity were significantly diminished (black arrowheads) (Figures 6C–E). At stage 20, the expression of *twist1* and *snai2* in the migrating NCC was also reduced upon Itpka KD (black arrowheads) (Figures 7A,B), which was confirmed by measuring the expression area (black dotted lines) (Figures 7C,D). The expression intensity was not altered (Figure 7E). At stage 23, the expression of the marker genes *twist1*, *foxd3* and *egr2* was decreased upon Itpka MO-injection (black arrowheads) (Figures 8A,B) as evidenced by a reduction in the expression area, intensity (black dotted lines) and length of the three branchial arches (black lines) (Figures 8C–H). In conclusion, Itpka depletion impairs NCC induction and migration, which may contribute to craniofacial malformations in *X. laevis* embryos.

### 2.4 Itpka is required for brain development in *X. laevis*

**FIGURE 9:**
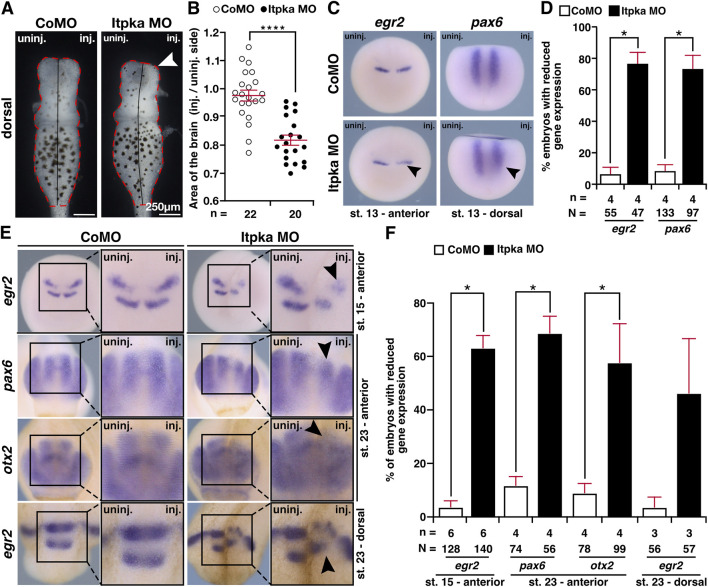
Itpka knockdown affects the development of the brain. **(A)** Dorsal view of isolated brains from control and Itpka morphants (stage 42). Smaller brain area on the injected side (white arrowhead). **(B)** Statistical evaluation of the brain area as indicated in **(A)**. **(C)** Anterior and dorsal view of control MO (CoMO)- and Itpka MO-injected embryos of *egr2* and *pax6* at stage 13 visualized by whole mount *in situ* hybridization (WMISH). Reduced expression is indicated by a black arrowhead. **(D)** Statistical evaluation of the brain-specific gene expression as illustrated in **(C)**. **(E)** Anterior and dorsal view of CoMO- and Itpka MO-injected embryos at stage 15 and 23 after WMISH with brain-specific genes *egr2, pax6* and *otx2.* Reduced gene expression on the Itpka MO-injected side in the mid- and hindbrain is indicated with black arrowheads. **(F)** Statistical evaluation of the brain-specific gene expression as illustrated in **(E)**. Abbreviations: CoMO, control morpholino oligonucleotide; *egr2*, *early growth response 2*; inj., injected; *itpka*, *inositol 1,4,5-trisphosphate 3-kinase A*; µm, micrometer; MO, morpholino oligonucleotide; n, number of independent experiments; N, number of analyzed embryos in total; *otx2*, *orthodenticle homeobox 2*; *pax6*, *paired box 6*; st., stage; uninj., uninjected; WMISH, whole mount *in situ* hybridization. Error bars indicate standard errors of the means: *, p ≤ *0.05;* ****, p ≤ 0.0001.

Previous studies have shown that Itpka loss of function negatively affects brain morphogenesis in adult mice (Köster et al., 2016). To analyze Itpka deficiency in *X. laevis* brain development, we isolated the brains of Itpka morphants (stage 42) and observed a significantly smaller brain area on the injected side (Figures 9A,B). Afterwards, we performed WMISHs with several brain-specific genes in different developmental stages of *X. laevis* embryos. At stage 13, where the induction of brain-specific cells starts, the expression of *egr2* (rhombomeres 3 and 4) and *pax6* (*paired box 6;* posterior neural tube) was significantly reduced upon Itpka MO KD (black arrowheads) (Figures 9C,D). Additionally, *egr2* showed a reduced expression at stage 15 (black arrowhead) (Figures 9E,F). At a later stage (stage 23) brain-specific genes such as *pax6* (forebrain and posterior neural tube), *otx2* (*orthodenticle homeobox 2*; forebrain and midbrain) and *egr2* (hindbrain) showed a reduced expression in Itpka MO-manipulated embryos in the fore-, mid- and hindbrain (black arrowheads) (Figures 9E,F). Taken together, these data indicate that Itpka depletion in very early stages affects brain cell induction and differentiation presumably contributing to brain defects in *X. laevis*.

### 2.5 Itpka is important for proper eye development in *X. laevis*

**FIGURE 10:**
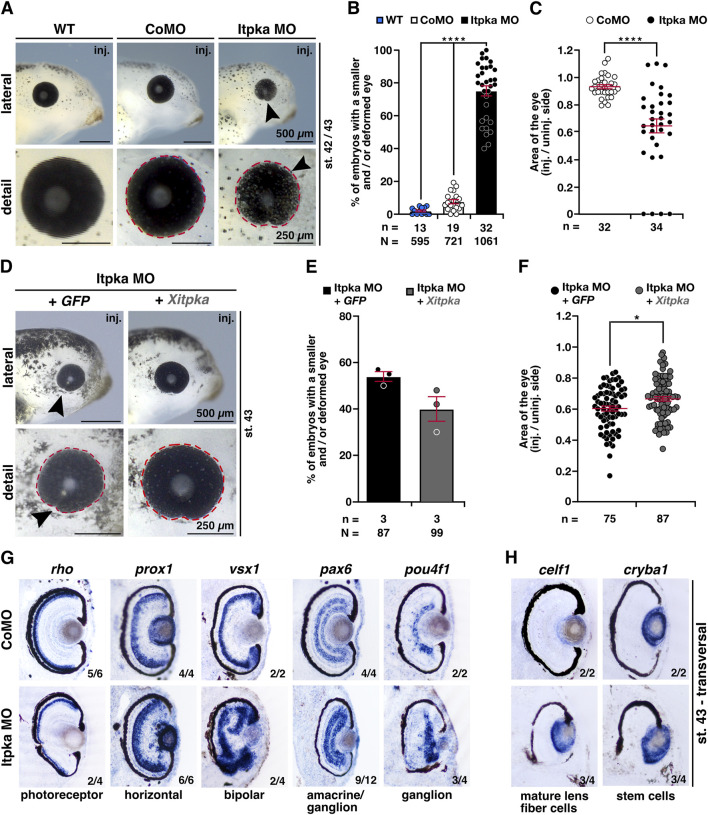
Itpka is required for eye development, while *Xitpka* rescues the Itpka MO-induced eye phenotype in *X. laevis*. **(A)** Lateral and detailed views of a representative wildtype, CoMO and Itpka MO-injected embryos at stage 42/43. Black arrowheads point to smaller eyes. **(B)** Statistical evaluation of smaller and deformed eyes as shown in **(A)**. **(C)** Statistical analysis of the eye area (red dashed lines) as illustrated in **(A)**. **(D)** Lateral and detailed views of Itpka MO-injected embryos in combination with *Xitpka*-RNA co-injection. Black arrowheads point to smaller eyes. Representative embryos at stage 43 are shown. **(E)** Statistical evaluation of smaller and deformed eyes as illustrated in **(D)**. **(F)** Statistical analysis of the eye area (red dashed line) as illustrated in **(D)**. **(G)** Transversal vibratome sections after whole mount *in situ* hybridization (WMISH) of Itpka MO-injected embryos at stage 43. Genes for specific cell populations of the retina are used as described in the main text. **(H)** Lens-specific genes *celf1* and *cryba1* are also affected. Abbreviations: *celf1*, CUGBP elav-like family member 1; CoMO, control morpholino oligonucleotide; *cryba1*, crystallin beta A1; *GFP*, green fluorescent protein; inj., injected; *itpka*, *inositol 1,4,5-trisphosphate 3-kinase A*; MO, morpholino oligonucleotide; n, number of independent experiments; N, number of analyzed embryos in total; *pax6*, paired box 6; *pou4f1*, POU class 4 homeobox 1; *prox1*, prospero homeobox 1; *rho*, rhodopsin; st., stage; *vsx1*, visual system homeobox 1; WT, wildtype; *Xitpka*, *Xenopus itpka*. Error bars indicate standard errors of the means: *, p ≤ 0.05; ****, p ≤ 0.0001.

As we investigated the head and brain phenotype, an eye phenotype was also noticeable. Itpka MO-injected embryos showed significantly smaller and/or deformed eyes on the injected side compared to WT and CoMO-injected embryos (Figures 10A,B). A detailed analysis of eye area (red dotted line) revealed a smaller eye size upon Itpka KD (Figures 10A,C). Itpka MO-mediated KD was partially rescued by co-injection of *Xitpka* RNA (Figures 10D,E). The detailed analysis of the eye area revealed that *Xitpka* significantly rescued the Itpka MO-induced eye phenotype (Figures 10D,F).

To investigate retinal lamination, we performed WMISHs of well-characterized retina-cell-type genes followed by transversal vibratome sectioning (Cizelsky et al., 2013). Most of the specific genes for retina cell layers such as the photoreceptor (*rho; rhodopsin*), horizontal (*prox1; prospero homeobox 1*), bipolar (*vsx1; visual system homeobox 1*), amacrine/ganglion (*pax6*) and ganglion (*pou4f1; pou class 4 homeobox 1*) cells displayed a disorganized localization and/or a smaller area in the retina upon Itpka MO KD (Figure 10G). Probes specific for the lens, *celf1* (*CUGBP elav-like family member 1*; mature lens fiber cells) and *cryba1* (*crystallin beta A1*; stem cells), show a diffuse expression in Itpka morphants (Figure 10H).

**FIGURE 11:**
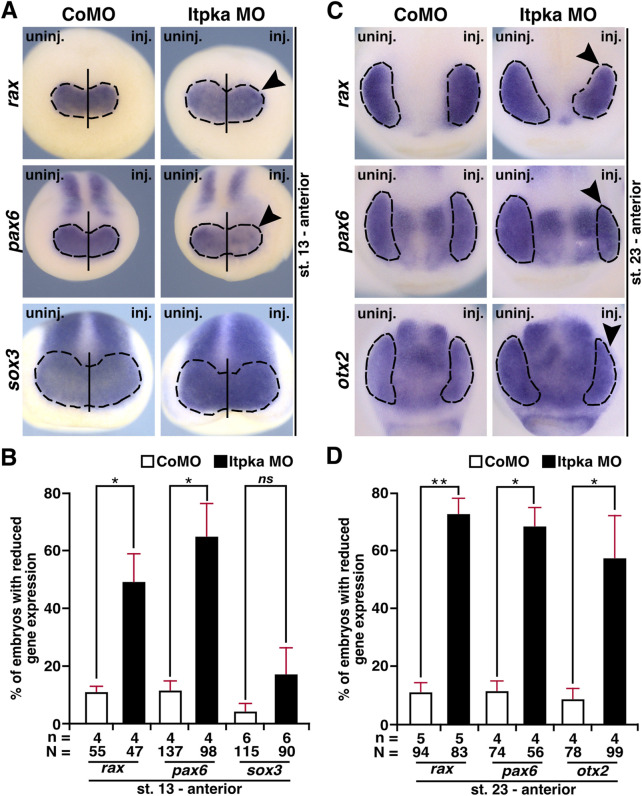
Itpka is necessary for proper differentiation of eye-specific genes in *X. laevis*. **(A)** Anterior expression (black dashed line) of the eye-specific genes *rax* and *pax6* as well as the pan-neural marker gene *sox3* in control and Itpka morphants at stage 13, during eye field induction, visualized by whole mount *in situ* hybridization (WMISH). Reduced expression is shown by black arrowheads. **(B)** Statistical evaluation of embryos with reduced gene expression as described in **(A)**. **(C)** Anterior view of CoMO- and Itpka MO-injected embryos illustrate eye-specific gene expression (black dashed line) of *rax*, *pax6*, and *otx2* at stage 23. Black arrowheads show a reduced expression in the developing eye. **(D)** Statistical evaluation of embryos with reduced gene expression as described in **(C)**. Abbreviations: CoMO, control morpholino oligonucleotide; inj., injected; *itpka*, *inositol 1,4,5-trisphosphate 3-kinase A;* MO, morpholino oligonucleotide; n, number of independent experiments; N, number of analyzed embryos in total; *ns*, non-significant; *otx2*, orthodenticle homeobox 2; *pax6*, paired box 6; *rax*, retina and anterior neural fold homeobox; *sox3*, SRY-box transcription factor 3; st., stage; uninj., uninjected; WMISH, whole mount *in situ* hybridization. Error bars indicate standard errors of the means. *ns*, p > 0.05; *, p ≤ 0.05; **, p ≤ 0.01.

To determine the molecular basis of the eye phenotype, we then investigated key steps of eye development. During eye field induction at stage 13, the anterior expression of the eye-specific genes *rax* (r*etina and anterior neural fold homeobox*) and *pax6* was significantly reduced (black arrowheads) in Itpka morphants at the injected side, while the expression of the pan-neural gene *sox3* (*sex-determining region Y-box 3*) remained unaffected (Figures 11A,B). At a later stage (stage 23) during eye cell differentiation, Itpka deficiency caused a reduction in the expression of all investigated eye cell-specific genes *rax*, *pax6*, and *otx2* (black arrowheads) (Figures 11C,D). Taken together, we have shown that Itpka is essential for proper eye development as well as eye cell differentiation and that its depletion already affects early eye field induction in *X. laevis*.

## 3 Discussion

### 3.1 *Itpka* conservation and expression in anterior neural tissue

In our study, *in silico* analysis of *ITPKA* identified strong evolutionary conservation across species. Consistent with previous findings that demonstrated a high degree of similarity of ITPKA and ITPKB in their C-terminal amino acid sequences and less conservation in the N-terminal region (Erneux et al., 1992), our data additionally show that there is a high homology of ITPKA protein alignment across species. Notably, this conservation is particularly strong within the C-terminal region, which includes the IPK and the calmodulin-binding regions.

To date, there is only one study in vertebrates that describes the expression kinetics of *itpka* during *X. tropicalis* development (Owens et al., 2016), showing that *itpka* is increased expressed from stage 10 on. Northern blot analysis revealed ubiquitous and strong expression of *Itpka* in the brain (especially in the hippocampus, neocortex and cerebellum) and testis of adult mice, but only a very low expression during early mouse embryogenesis. This expression increased with postnatal development to adulthood (Mailleux et al., 1993; Vanweyenberg et al., 1995). In our study, we observed an enrichment of transcripts in neuroectoderm-derived structures such as the cells of neural crest, brain and retina starting at stage 10. At the transcriptional level, *itpka* has been detected in tissues of the anterior neural head region and the eye during early embryonic development of *X. laevis*. These findings confirm a mild expression in the developing anterior neural structures during embryogenesis and indicate a potential function of Itpka in the development of the central nervous system of *X. laevis*. Since we only investigated embryonic stages, we could only speculate about the expression levels in the metamorphosis of *X. laevis* and the adult frog.

### 3.2 Itpka depletion causes defects in anterior neural structures and affects crucial steps of their development

Our functional analysis using Itpka MO-mediated KD in *X. laevis* revealed severe developmental defects in anterior structures, particularly affecting the head, brain and retina. These Itpka morphants displayed microcephaly, cranial cartilage and nerve defects, as well as microphthalmia, accompanied by disrupted retinal lamination and altered lens morphology. These severe phenotypes were successfully rescued by co-injection of *X. laevis itpka* RNA, confirming the validity of our model for studying the functional role of Itpka in vertebrate development.

Itpka has a bifunctional activity, being involved in actin bundling and calcium signaling (Schell et al., 2001; Windhorst et al., 2012) – both molecular processes are crucial for neuronal development and function. Regarding actin, expression of Itpka in hippocampal neurons leads to an elongation of dendritic spines (Köster et al., 2016) while the overexpression of the full protein in primary neurons results in an increase of dendritic spine number (Windhorst et al., 2012). To our knowledge the severe developmental defects in anterior neural structures that we described were not investigated or shown in mice or rats (Jun et al., 1998). It is also reported that retardation of spine development in young mice could recover with age (Kim et al., 2009), therefore it would be interesting to investigate adult Itpka KO frogs. Moreover, the lack of information about Itpka KO mouse embryos gives rise to further investigations in early mouse embryogenesis.

Calcium signaling regulates various cellular processes including metabolism, cell proliferation, and brain development (Berridge, 2016). Additionally, phospholipase C, which initiates the InsP_3_/Ca^2+^ signaling pathway that is regulated by Itpka, plays a crucial role in brain development (Kang et al., 2016). In line with the aforementioned involvement of Itpka in crucial processes regulating neuronal development and morphology, our study shows that Itpka MO KD leads to a significant impairment of brain development. This aligns with previous findings in adult *Itpka* knockout mice and rats, which show both neuromorphological (Köster et al., 2016) and neurobehavioral abnormalities (Blechner et al., 2020). Interestingly, such findings are commonly observed in neurodevelopmental disorders like schizophrenia (Takahashi et al., 2011) or autism spectrum disorder (ASD).

To explore the reason for smaller head structures and eyes, we investigated cell proliferation and apoptosis, but neither pH H3 nor TUNEL staining was altered in the anterior neural region of the head compared to the control embryos at stage 23. These results indicate that the observed changes are not caused by altered or abnormal cell proliferation or apoptosis measured with these methods at this stage.

This prompted us to further investigate the molecular basis of the developmental phenotypes observed and analyzed the expression of several key neurodevelopmental genes upon Itpka KD. Given that embryonic development involves coordinated processes such as cell differentiation and migration, we focused on genes associated with neural crest cell (NCC), eye, and brain development.

NCCs contribute to a wide range of cell types and structures, including craniofacial skeleton, peripheral nervous system, and ocular tissues (Le Douarin and Dupin, 2018). Previous studies have shown that abnormalities in NCC development can result in craniofacial defects and ocular anomalies (Siismets and Hatch, 2020; Williams and Bohnsack, 2015). In line, aberrant NCC development and differentiation give rise to a group of severe disorders known as neurocristopathies. Patients with these conditions present a phenotype comparable to those observed in Itpka KD *X. laevis* embryos, including craniofacial abnormalities such as malformed skull bones, cleft palate and visual impairments (Vega-Lopez et al., 2018). Our data demonstrate that Itpka KD led to a significant reduction in the expression of key NCC genes during cell induction, differentiation and migration, including *snai2*, *egr2*, *twist1*, and *foxd3*. This downregulation and altered migration likely contribute to the craniofacial and cranial nerve defects observed. Furthermore, transcriptional regulators such as *snai2* or *foxd3* are known to play a crucial role in neural differentiation, with mutations leading to impaired neurogenesis (Méndez-Maldonado et al., 2020). In summary, the decreased expression of NCC genes upon Itpka KD may represent a fundamental impact on the molecular basis underlying the developmental defects we observed.

We further examined genes essential for eye development, such as *rax* and *pax6*, and observed reduced expression during early eye field induction and cell differentiation upon Itpka KD. Specifically, the downregulation of *rax*, a gene required for retinal progenitor cell proliferation and cell fate specification, contributes to retinal malformations (Rodgers et al., 2018). Decreased expression of *PAX6*, *OTX2*, and *EGR2*, which are all associated with human brain and eye defects (Deml et al., 2016; Gonzalez-Rodriguez et al., 2010; Nakayama et al., 2015; Sevilla et al., 2015), further supports the relevance of our findings. Interestingly, expression of the pan-neural gene *sox3* remained unchanged at stage 13, indicating that early neural induction is not broadly disrupted by Itpka KD.

Taken together, our results show that Itpka KD leads to a reduced expression of key developmental genes, resulting in severe head, brain, and eye abnormalities. Based on our findings, we propose that Itpka plays an essential role in early development by possibly regulating calcium signaling and/or actin dynamics, both crucial for cellular processes that shape the anterior nervous system. Further investigation into the precise mechanisms of Itpka function during central nervous system development is essential.

## 4 Materials and methods

### 4.1 Synteny analysis and protein alignment of itpka

Synteny analysis of *itpka* was performed by comparing the gene location between the species: human, mouse, frog, and fish using the NCBI GenBank (https://www.ncbi.nlm.nih.gov/genbank/). To determine the protein regions within Itpka such as the regions required for cytoskeleton location, IPK and calmodulin binding in the different species, the NCBI GenBank was used. Afterwards, multiple sequence alignments and calculations of the homology from Itpka were done using the ClustalW and Clustal Omega multiple sequence alignment tools from the EMBL-EBI homepage. The following sequences from the NCBI GenBank were used: *Homo sapiens*: NP_002211.1; *Mus musculus*: NP_666237.1; *Xenopus laevis* S: XP_018087711.1; *Xenopus laevis* L: XP_018086619.1; *Xenopus tropicalis*: XP_002935396.3; and *Danio rerio*: NP_001313405.1.

### 4.2 *Xenopus laevis* embryos

*X.laevis* embryos were generated, cultured, and staged according to the standard protocols (Nieuwkoop and Faber, 1994; Sive et al., 2010). All experimental procedures were performed in agreement with the German animal use and care law. Furthermore, *in vivo* experiments were approved by the administration of the state of Baden-Württemberg (Regierungspräsidium Tübingen). Embryos were cultivated in 0.1× modified Barth’s saline with HEPES buffer (MBSH) at 12.5°C–20°C and fixed with 1× MEMFA(T) [0.1 M MOPS, pH 7.4; 2 mM EGTA, 1 mM MgSO4 (H2O)7, 4% formaldehyde, 0.1% Tween] at the desired stage.

### 4.3 Whole mount *in situ* hybridization (WMISH) and histology

To study the spatio-temporal expression profile during *X. laevis* embryogenesis, whole mount *in situ* hybridization (WMISH) analysis was performed according to the established protocols (Hemmati-Brivanlou et al., 1990; Lufkin, 2007). Digoxigenin (DIG)-labeled antisense RNA probes were generated by *in vitro* transcription using T7, SP6, or T3 RNA polymerase (Roche, Basel, Switzerland). Subsequently, the embryos were stained with BM Purple (Roche, Basel, Switzerland) for up to 14 days for exterior view or NBT/BCIP (Roche, Basel, Switzerland) for up to 14 days for sectioning. BM Purple-stained embryos were bleached with 30% H_2_O_2_. For more detailed tissue analysis, NBT/BCIP-stained or wildtype *X. laevis* embryos were equilibrated in 1 mL gelatin/albumin solution (2.2 g gelatin, 135 g BSA, 90 g sucrose, and 500 mL 1× PBS) overnight at 4°C and embedded in 1 mL gelatin/albumin solution with 75 μL glutaraldehyde (Fluka, Switzerland). Using a vibratome (Vibratome 1500 Classic, The Vibratome Company), we made sections with a thickness of 25 μm according to (Guo et al., 2011). For investigating the spatio-temporal expression pattern of *itpka* during *X. laevis* development, we cloned cDNA fragments of 1151 bp (*itpka.L*, part of 5′UTR and coding sequence) with the cloning primers itpka_l 5′-GAG AAA GGA GGA AGT GAG-3′ and itpka_r 5′-AAG GTT GAG GTT GAA CTG-3′, into the pSC-B vector (Stratagene, La Jolla, California, United States). For all PCR amplifications, we used cDNA from stage 28 of *X. laevis* embryos and the proof reading PfuUltraTM II fusion HS DNA polymerase (Agilent Tech., Santa Clara, California, United States). We used the following RNA antisense probes as described previously: *celf1* (CUGBP elav-like family member 1) (Day and Beck, 2011; Rothe et al., 2017), *cryba1* (crystallin beta A1) (Day and Beck, 2011), *egr2* (early growth response 2) (Cizelsky et al., 2013), *foxd3* (forkhead box D3) (Gessert et al., 2007; Li et al., 2009), *itpka* (inositol-) (this publication), *otx2* (orthodenticle homeobox 2) (Lamb and Harland, 1995), *pax6* (paired box 6) (Hitchcock et al., 1996; Hollemann et al., 1998), *pou4f1* (pou class 4 homeobox 1) (Liu et al., 2000), *prox1* (prospero homeobox 1) (Dyer et al., 2003), *rax* (retina and anterior neural fold homeobox) (Furukawa et al., 1997), *rho* (rhodopsin) (Chang and Harris, 1998), *snai2* (snail family zinc finger 2) (clone ID: pMX363), *sox3* (sex-determining region Y-box 3) (Maurus et al., 2005), *twist1* (twist family bHLH transcription factor 1) (Gessert et al., 2007) and *vsx1* (visual system homeobox 1).

### 4.4 RNA isolation and RT-PCR assay

To analyze the temporal expression of *itpka*, *X. laevis* embryos were collected, tissues were isolated as indicated and fixed by freezing at −80°C at different developmental stages (13, 23, 32, 42). The total RNAs were isolated from *X. laevis* embryos using the peqGOLD RNAPure Kit (Peqlab, Erlangen, Germany) by following the manufacturer’s protocol. cDNA was generated using random primers and the SuperScript II reverse transcriptase (Invitrogen, Carlsbad, California, United States). RT-PCRs were performed with the same set of cDNA using the Phire Hot Start II DNA polymerase (Thermo Scientific, Waltham, Massachusetts, United States).

### 4.5 Morpholino oligonucleotides (MO), cloning, injection mRNA, and microinjections

To perform knockdown (KD) experiments, the Itpka morpholino oligonucleotide (MO) was designed to the sequence of *itpka* L and S homologue: 5′-CAT CCA AAA CAC AAA GCT GCG GG-3′. Itpka MO and the standard control morpholino oligonucleotide (CoMO) (5′-CCT CTT ACC TCA GTT ACA ATT TAT A-3′) were purchased from Gene Tools (Philomath, OR, United States). The MOs were diluted in autoclaved diethyl-pyro-carbonate (DEPC)-treated water. To target anterior neural tissue, 15 ng of Itpka MO was unilaterally injected (inj.) into one animal-dorsal blastomere of eight-cell-stage *X. laevis* embryos (Moody and Kline, 1990). The uninjected (uninj.) side served as an internal control. As injection control, CoMO was injected (15 ng). Successful and correct injections were controlled by the co-injection of 0.4–0.5 ng RNA coding for *GFP*. For rescue experiments, *X. laevis Δ-5′UTR-itpka-l* (*Xitpka*) RNA was cloned into the pCS2+ vector (Rupp and Weintraub) using *StuI* for restriction and the following primers: *Δ5′UTRitpka-l* 5′-AGG CCT ATG ATT CCT ACG GAA GTT GCA-3′ and *Δ5′UTRitpka-r* 5′-AGG CCT TCC TAG TGT AGT GGT CAG TAA-3’. Generation of injection mRNA was performed by *in vitro* transcription using SP6 and T7 polymerase. Rescue experiments of Itpka MO were accomplished by injection of 10 ng Itpka MO with 0.25 ng *Xitpka* RNA co-injection into one animal-dorsal blastomere of an 8-cell-stage embryo. *Xitpka* cannot target Itpka MO because of the altered sequence as shown in Supplementary Figure S2A. To adjust the amount of RNA per injection, *GFP* RNA were used for all experiments.

### 4.6 Morpholino oligonucleotide (MO) binding efficiency

To show the specificity of the Itpka MO to its corresponding Itpka MO binding site a MO binding efficiency test was performed. We cloned the MO binding site of Itpka MO and the binding site of the rescue *Xitpka* in front and in frame with the green fluorescent protein (*GFP*) gene. Following cloning primers were used: *itpka-MO-bs_l* 5′-GA TCC CCC CGC AGC TTT GTG TTT TGG ATG GGG-3′, *itpka-MO-bs_r* 5′-AA TTC CCC ATC CAA AAC ACA AAG CTG CGG GGG-3′, *Δ-itpka-MO-bs_l* 5′-GA TCC ATC GAT TCG AAT TCA AGG CCT ATG GGG-3′ and *Δ-itpka-MO-bs_r* 5′-AA TTC CCC ATA GGC CTT GAA TTC GAA TCG ATG-3’. To test the Itpka MO binding efficiency, either 10 ng of CoMO or Itpka MO was co-injected with 1 ng of the respective fusion construct RNA into two-cell stage embryos, and the successful or blocked translation of the green fluorescent protein (GFP) was monitored at stage 20–25.

### 4.7 Cartilage staining by alcian blue and cranial nerve staining by 3A10 antibody

In order to investigate the craniofacial cartilage and cranial nerves, embryos injected with 15 ng Itpka MO and CoMO were fixed at late tadpole stages. Alcian blue staining: embryos were stained with Alcian blue as previously described (Gessert et al., 2007), and afterwards, the cranial cartilage was dissected and imaged. 3A10 antibody staining: embryos were treated with the monoclonal 3A10 antibody (DSHB, Iowa City) to visualize the cranial nerves (Schuff et al., 2007).

### 4.8 Phospho-histone 3 staining and TUNEL assay

Proliferative cells of stage 23 *X. laevis* embryos were stained for phospho-histone H3 (pH H3) and apoptotic cells of stage 23 *X. laevis* embryos were stained with TUNEL according to the established protocols (Cizelsky et al., 2013; Gessert et al., 2007).

### 4.9 Imaging

Representative embryos/experiments were imaged. Whole *X. laevis* embryos from the exterior view were imaged by using an Olympus MVX10 (fluorescence) and an Olympus UC75 camera. Vibratome sections were imaged with an Olympus RX60 microscope and an Olympus DP28 camera. Images were processed with ImageJ2 version 2.9.0 (Rueden et al., 2017) and Affinity Designer 1.10.8.

### 4.10 Quantitative tissue measurements

For all quantitative measurements, one/two representative experiments of unilaterally injected control MO (CoMO) and Itpka MO embryos were used. The area and width of the head as well as the eye area were measured using the software ImageJ2 version 2.9.0 (Rueden et al., 2017). For brain area measurements, the brains of stage 42 embryos were dissected and imaged. ImageJ2 (Rueden et al., 2017) was used to measure the area of the brain as well as the expression area and intensity of the different marker genes. The values (injected/uninjected side) were then calculated and analyzed using GraphPad Prism 10.4.0 for macOS (Boston, Massachusetts, United States) as well as the expression area and intensity of the different marker genes.

### 4.11 Statistics

Data were analyzed with GraphPad Prism 10.4.2 for macOS (Boston, Massachusetts, United States, www.graphpad.com). Only experiments with a higher survival rate than 50% were considered for statistical evaluation. Statistical evaluation was performed only with more than three independent experiments. To determine statistical differences, the non-parametric Mann-Whitney rank-sum test was used, and the error bars represent the standard errors of the mean (SEM). Statistical significances are indicated as ns, p > 0.05; *, p ≤ 0.05; **, p ≤ 0.01; ***, p ≤ 0.001; and ****, p ≤ 0.0001.

## Data Availability

The original contributions presented in the study are included in the article/Supplementary Material, further inquiries can be directed to the corresponding author.

## Supplementary material

SUPPLEMENTARY FIGURE S1 Gene specific RNA-antisense probe from *itpka* binds specifically to the complementary sense *itpka* mRNA using WMISH. Staining conditions for sense and antisense probes were identical. Embryonic stages and scale bars are indicated in each panel. Scale bars are equivalent to 500 μm. Stage 4 animal view. Stage 10 vegetal view. Stage 13 dorsal view. Stage 23, 28, 36 and 40 lateral views.Abbreviation: st., stage; *itpka*, *inositol 1,4,5-trisphosphate 3-kinase A*; µm, micrometer.

SUPPLEMENTARY FIGURE S2 Itpka MO specifically binds *X. laevis itpka* mRNA. **(A)** MO binding sites (MObs) of 5′UTR *Xenopus itpka* (5′UTR *Xitpka*) and Δ5′UTR *Xenopus itpka* (Δ5′UTR *Xitpka*) are indicated. The ATG start codon is highlighted in blue and the differing bases of the *itpka* Mo*bs-GFP* and Δ5′UTR *Xitpka Mobs-GFP* are shown in grey. MObs were recombined in front of and in frame with *GFP*. **(B)** The *GFP* fusion RNA was co-injected together with Itpka or control MO (CoMO) at the 2-cell-stage and monitored at stage 20. CoMO and Itpka MO are shown. The percentage of GFP-positive embryos are given. Abbreviations: binding sites (bs), CoMO, control morpholino oligonucleotide; *GFP*, green fluorescent protein; *itpka*, *inositol 1,4,5-trisphosphate 3-kinase A;* MO, morpholino oligonucleotide; UTR, untranslated region; *Xitpka, Xenopus itpka*.

SUPPLEMENTARY FIGURE S3 Itpka knockdown leads neither to a change in proliferative nor apoptotic cells in anterior neural development. **(A)** Proliferative cells of embryos injected with control MO (CoMO) or Itpka MO were stained with a phospho-histone H3 (pH H3) antibody at stage 23. The anterior view of embryos was imaged and the number of proliferative cells were counted. The area of the anterior neural plate of the injected and uninjected side were compared. The black boxes indicate the area where proliferative cells were counted. **(B)** Statistical evaluation of the number of pH H3 positive cells as illustrated in **(A)**. **(C)** Apoptotic cells of embryos injected with CoMO or Itpka MO were stained with a TUNEL (terminal deoxynucleotidyl transferase dUTP nick end labeling) antibody at stage 23. The anterior view of embryos was imaged and the number of apoptotic cells were counted. The area of the anterior neural plate of the injected and uninjected side were compared. The black boxes indicate the area where apoptotic cells were counted. **(D)** Statistical evaluation of the number of TUNEL positive cells as illustrated in **(C)**. Abbreviations: CoMO, control morpholino oligonucleotide; *itpka*, *inositol 1,4,5-trisphosphate 3-kinase A*; inj., injected; MO, morpholino oligonucleotide; n, number of independent experiments; N, number of analyzed embryos in total; *ns*, non-significant; pH H3, phospho-histone H3; TUNEL, terminal deoxynucleotidyl transferase dUTP nick end labeling; uninj., uninjected. Error bars indicate standard errors of the means: *ns, p* > *0.05.*
