# Supplementary figures and images for: Itpka depletion implicates defects in anterior neural development of Xenopus laevis
Source: Front Cell Dev Biol. 2025 Jul 9;13:1610183. doi: 10.3389/fcell.2025.1610183 (PMC12283734; doi:10.3389/fcell.2025.1610183)

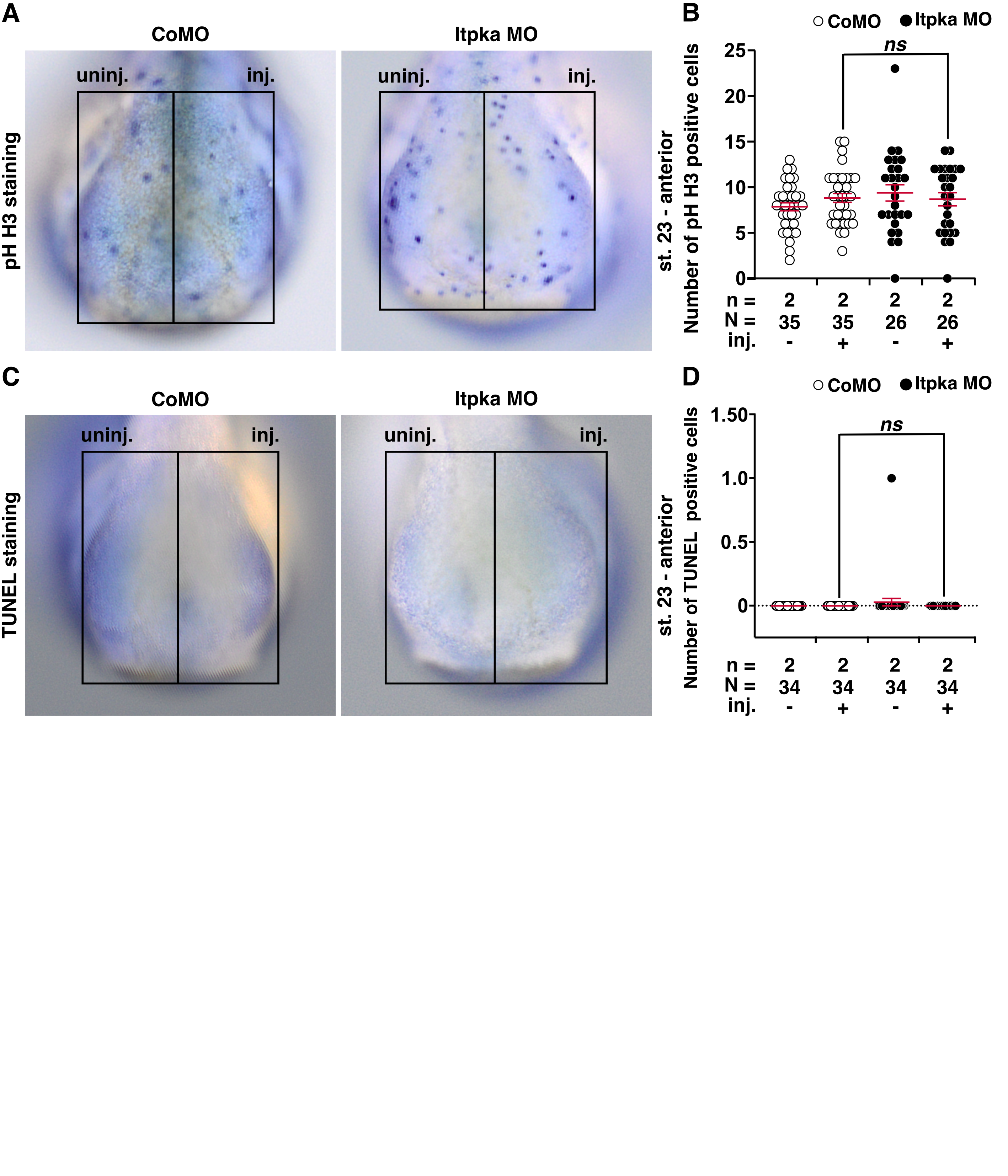

Supplement: Supplementary file 1 [file Image3.tiff]

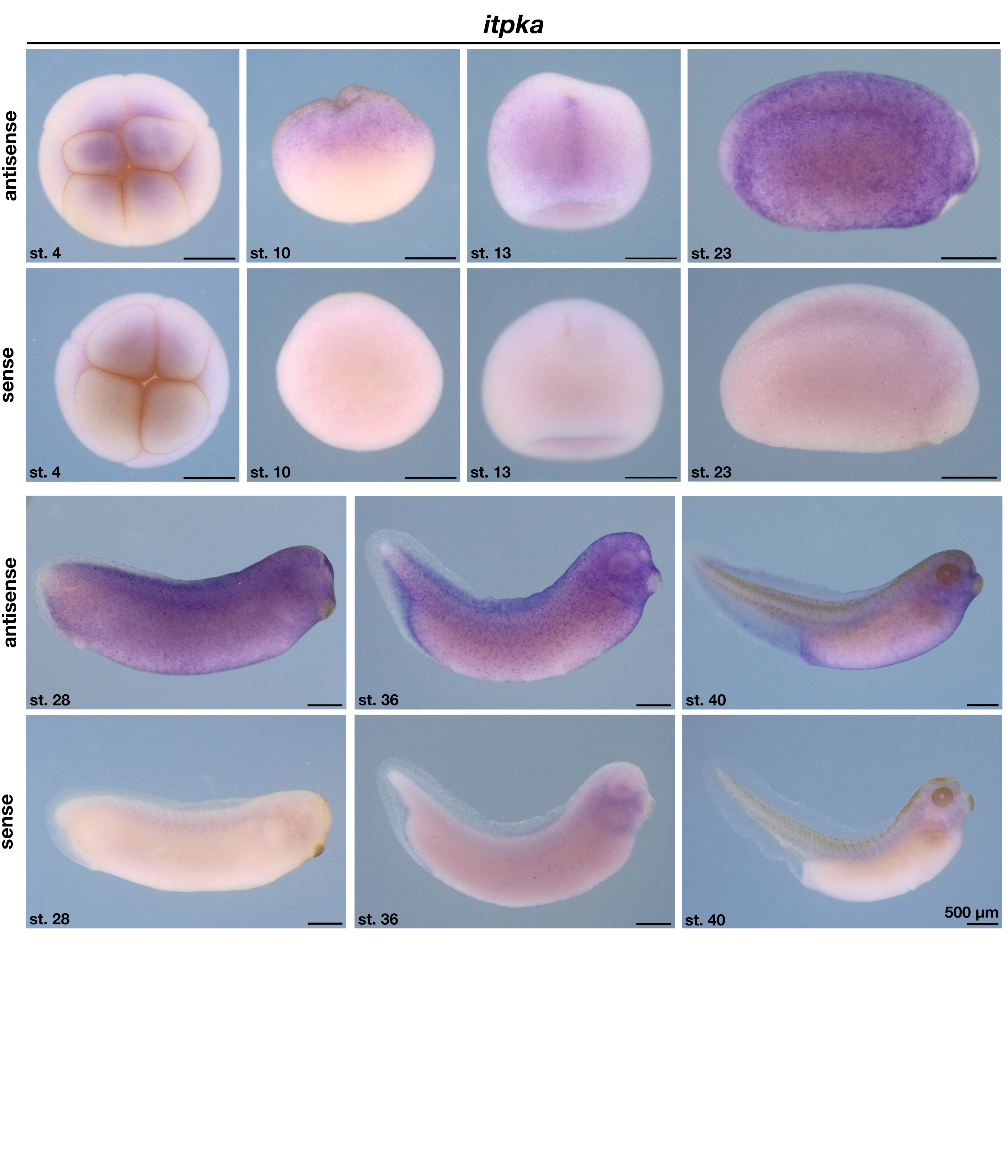

Supplement: Supplementary file 2 [file Image1.tiff]

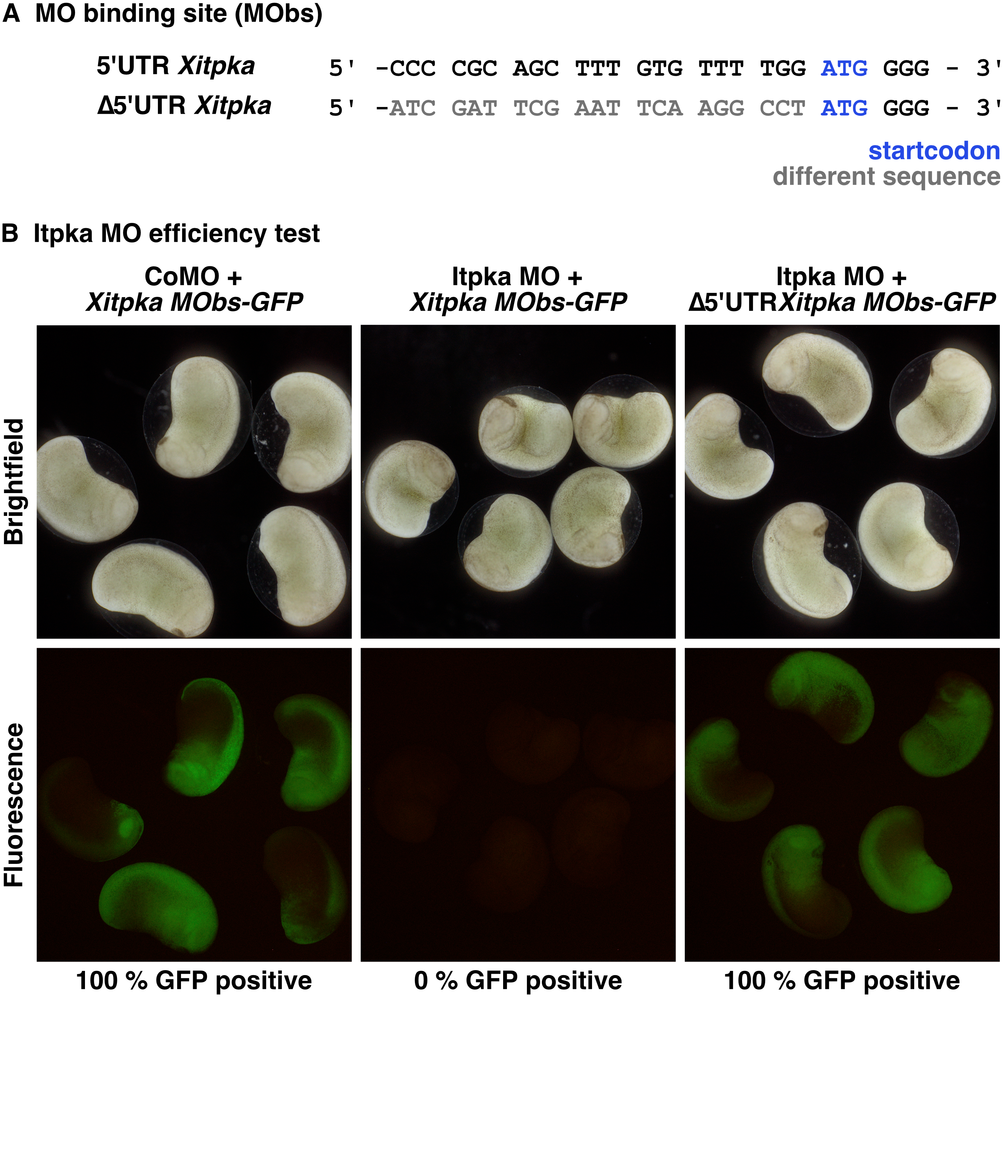

Supplement: Supplementary file 3 [file Image2.tiff]
